# Risk factors associated with weight gain after kidney transplantation: A cohort study

**DOI:** 10.1371/journal.pone.0243394

**Published:** 2020-12-28

**Authors:** Cristina Carra Forte, Elis Forcellini Pedrollo, Bruna Bellincanta Nicoletto, Jéssica Blatt Lopes, Roberto Ceratti Manfro, Gabriela Corrêa Souza, Cristiane Bauermann Leitão

**Affiliations:** 1 Post-graduate Program in Medical Sciences: Endocrinology, School of Medicine, Universidade Federal do Rio Grande do Sul Faculdade de Medicina, Porto Alegre, Brazil; 2 Universidade Federal do Rio Grande do Sul, Porto Alegre, Brazil; 3 Life Science Knowledge Area, Universidade de Caxias do Sul, Caxias do Sul, Brazil; 4 Nutrition Graduate Course, School of Medicine, Universidade Federal do Rio Grande do Sul, Porto Alegre, Brazil; 5 Post-graduate in Medicine: Medical Sciences, Universidade Federal do Rio Grande do Sul, Porto Alegre, Brazil; 6 Division of Nephrology, Hospital de Clínicas de Porto Alegre, Porto Alegre, Brazil; 7 Division of Nutrition, Hospital de Clinicas de Porto Alegre, Porto Alegre, Brazil; 8 Division of Endocrinology, Hospital de Clínicas de Porto Alegre, Porto Alegre, Brazil; San Raffaele Roma Open University, ITALY

## Abstract

**Background:**

Renal transplantation is the best modality of renal replacement therapy for patients with end-stage renal disease. However, it is associated with weight gain and metabolic abnormalities, which adversely impact transplant outcomes.

**Objective:**

The objective of this study was to identify the risk factors of one-year weight gain after renal transplantation.

**Methods:**

A retrospective cohort study was conducted with 374 patients that underwent kidney transplantation between January 2006 and July 2013. Clinical and laboratory variables were collected from electronic records, and the outcome of interest was weight gain during the first year after renal transplantation. The data were reported as mean ± standard deviation, median (interquartile range) or number of subjects (%). The association between variables were assessed via chi-square test and ANOVA. For analysis of risk factors related to the outcomes of interest, multivariable logistic regression models were used.

**Results:**

There were 181 (48.4%) female patients, 334 (89.3%) with white ethnicity and the mean age was 44.4 ± 12.8 years. The mean BMI pre-transplant was 24.7 ± 4.1 kg/m^2^, and 35 (9.9%) patients were classified as obese; 119 (33.6%) as overweight; 187 (52.8%) as normal weight; and 13 (3.7%) as malnourished. After one year of follow-up, the mean BMI was 26.2 ± 5.0 kg/m^2^, and 61 (17.3%) patients were classified as obese; 133 (37.8%) as overweight; 148 (42.0%) as normal weight; and 10 (2.8%) as malnourished. Weight gain was observed in 72.7% patients, and the average increase was 7.12 ± 5.9 kg. The female gender, lower pre-transplant body weight, lower number of hospitalizations, and a kidney received from a living donor were associated with weight gain by more than 5% in the first year post-transplant.

**Conclusion:**

Female gender and lower pre-transplant body weight were independently associated with weight gain by more than 5% in the first year after kidney transplantation; lower rates of hospitalization and donation from living donors were also risk factors for this outcome.

## Introduction

Renal transplantation is considered the best modality of renal replacement therapy for patients with end-stage renal disease, as it enhances quality of life and patient survival when compared to other therapies, such as hemodialysis and peritoneal dialysis [1–3]. The number of solid organ transplants is increasing over the decades, mainly due to a rise in the amount of kidney transplants [4].

Faced with increased graft and patient survival after renal transplantation in recent decades, concerns have been raised regarding the quality of life of renal transplant recipients. Several factors have been associated with increased cardiovascular risk in renal transplant recipients during the post-operative period, such as the development of post-transplant diabetes mellitus (PTDM), hypertension, dyslipidemia and obesity [5–7]. Approximately 50% of these patients gain weight after renal transplantation, regardless of pre-transplant nutritional status [8–10]. In our center of Nephrology of the Hospital de Clínicas de Porto Alegre, patients submitted to renal transplantation gain a mean of 2.9 kg within one year of follow-up and 6.5 kg after five years, which represents 5.1% and 10.6% of original body weight, respectively [11, 12]. Factors such as ethnicity, correction of uremia, use of corticosteroids, increased food intake and sedentary lifestyle may be involved in the increase in body mass index (BMI) after renal transplantation [13–18].

Increased body weight and its negative metabolic consequences may be associated with worse outcomes after renal transplantation [19]. In a recent systematic review and meta-analysis, diagnosis of metabolic syndrome after renal transplantation was associated with an increased risk of graft loss, cardiovascular mortality and all-cause mortality [19]; therefore, the identification of factors associated with weight gain in renal transplant patients may contribute to the implementation of preventive and therapeutic measures to avoid weight increase and its consequences. Thus, the aim of this study was to identify the risk factors of one-year weight gain after renal transplantation.

## Patients and methods

This retrospective cohort study assessed renal transplant patients attending the Nephrology Division of the Hospital de Clínicas de Porto Alegre, Rio Grande do Sul, Brazil, from January 2006 to July 2013. The study was approved by the Research Ethics Committee and the Animal Use Ethics Committee of Hospital de Clínicas de Porto Alegre (protocol no. 130051), in accordance with the ethical standards for human research set forth in the Declaration of Helsinki [20]. All researchers involved signed the Term of Commitment for the use of data for research purposes, maintaining confidentiality of information from the patient registry.

The following exclusion criteria were used: age less than 18 years old; incomplete weight data pre-transplant or at 12 months post-transplantation; transplantation of multiple organs; less than one year of follow-up after transplantation; pre-transplantation diabetes mellitus (DM; diagnosed by the American Diabetes Association criteria) [21]; re-transplantation; graft lost; and death in the first year after transplantation.

Data were retrospectively collected, and a structured questionnaire evaluated the following variables: age at transplant; gender; ethnicity; pre-transplant dry weight; etiology of renal disease; dialysis type (hemodialysis or peritoneal dialysis) and duration; donor type (living or deceased) and gender; presence of HCV or cytomegalovirus (CMV) antibodies; diagnosis of delayed graft function (DGF); and development of PTDM. The cumulative dose was calculated as the sum of corticoid dosages during the first year of follow-up, divided by 365 days; thus, representing the mean daily dose during the first year. The estimated glomerular filtration rate (eGFR) was calculated using the data equation from the *Diet Modification in Kidney Disease* (MDRD) study, with data on serum creatinine, age, ethnicity and gender variables, in formula eGFR (mL/min/1.73 m^2^) = 186 × (serum creatinine)^1.154^ × (age)^0.203^ × 1.212 (African American) × 0.742 (female) or 1.0 (male) [22]. Pre-transplant information was collected by contacting the 38 dialysis centers were patients were treated.

Anthropometric evaluation consisted of body weight (in kg) and height (in m) measurements, with patients wearing light clothes without shoes, and BMI (calculated as the weight (kg)/height^2^ (m) ratio) [23]. Weight was assessed pre-transplant (dry weight being the weight at the end of a hemodialysis session without extra fluids in the body), at 3, 6 and 12 months post-transplant, as the weight gain is greater in the first 12 months. The BMI classification was based on cut-off points proposed by the World Health Organization [23]: low weight (BMI <18.5 kg/m^2^); normal weight (BMI = 18.5–24.9 kg/m^2^); overweight (BMI 25.0–29.9 kg/m^2^); obesity grade I (BMI 30.0–34.9 kg/m^2^); obesity grade II (BMI 35.0–39.9 kg/m^2^) and obesity grade III (BMI ≥ 40.0 kg/m^2^).

Lipid profiles (total cholesterol, HDL-cholesterol and triglycerides) and fasting glucose measurements, available at dialysis centers from the pre-transplant period, were collected by medical chart reviews. Post-transplant information was collected by review of electronic charts.

Our sample power calculation was performed with STATA software, based on the study by Cashion et al. [15], which evaluated demographic, clinical and environmental factors associated with weight gain during the post-transplant period. In that study, the sample size was 96 patients and a 2.3 kg difference in pre-transplant and post-transplant weight was observed. Our sample of 374 patients, was powered to detect the difference found (pre-transplant weight 67 kg ± 12.7 and post-transplant 71.1 kg ± 13.9), considering an alpha of 0.05 with a beta error of 0.988 (power rounded to 100%).

Data were analyzed using the statistical software package *Statistical Package for the Social Sciences* (SPSS Inc., version 22.0 for Windows, Chicago, IL, USA) and were reported as mean ± standard deviation, median (interquartile range) or number of subjects (%) with the specified variable. The association between categorical variables was assessed by chi-square test, and the differences between continuous variables were evaluated by ANOVA. For analysis of risk factors related to the outcome of interest (body weight gain >5%), multivariable logistic regression models were used, and the independent variables were chosen based on unadjusted results from ANOVA analysis (Table 1). Three models were used, as some variables may be collinear. Statistical significance was set at P < 0.05 for all analyses.

**Table 1:** Baseline patient characteristics, according to body weight change one year after kidney transplantation.

Variables	Total (n = 374)	Weight loss >5% (n = 48)	Weight change between <5% and >5% (n = 131)	Weight gain >5% (n = 195)	P
Patient related					
Age*	44.4 ± 12.8	47.6 ± 13.8^a^	46.2 ± 12.4^a^	42.4 ± 12.5^b^	<0.01
Female gender†	181 (48.4)	24 (50.0) ^ab^	47 (35.9) ^a^	110 (56.4) ^b^	<0.01
White†	334 (89.3)	44 (91.7)	117 (89.3)	173 (88.7)	0.75
Pre-transplant weight, kg*	67 ± 12.7	69.2 ± 10.9^a^	70.4 ± 13.2^a^	64.2 ± 12.2^b^	<0.01
Weight at12 months, kg*	71.1 ± 13,9	61.8 ± 10.4^a^	71.0 ± 13.5^b^	73.5 ± 14.1^b^	<0.001
Pre-transplant BMI, kg/m^2^*	24.7 ± 4.1	25.9 ± 3.4^a^	25.1 ± 3.9^ab^	24.2 ± 4.3^b^	0.01
BMI at 12 months, kg/m^2^*	26.2 ± 5.0	23.2 ± 3.4^a^	25.3 ± 4.1^b^	27.7 ± 5.5^c^	<0.01
Overweight patients*	119 (33.6)	28 (60)^a^	48 (39)^ab^	43 (23)^b^	<0.001
Obesity patients*	35 (9.9)	4 (8.5)^a^	13 (10.5)^a^	18 (9.8)^a^	>0.05
Hypertension	106 (28.3)	17 (35.4)	39 (29.8)	50 (25.6)	0.06
HCV†	33 (8.8)	06 (12.5)	10 (7.6)	17 (8.7)	0.59
CMV†	74 (19.8)	10 (20.8) ^a^	36 (27.5) ^a^	28 (14.4) ^b^	0.01
Biochemical tests, mg/dL					
Total Cholesterol pre-transplant*	170.1 ± 42.5	176.3 ± 46.5	174.0 ± 43.4	165.2 ± 40.6	0.29
HDL-cholesterol pre-transplant*	40.5 ± 12.9	40.8 ± 14.7	38.9 ± 12.9	38.9 ± 12.9	0.38
LDL-cholesterol pre-transplant*	99.6 ± 33.8	100.7 ± 32.8	99.9 ± 33.9	98.9 ± 34.4	0.97
Triglycerides pre-transplant**	169.4 ± 86.8	146.5 (107.0–230.0)	172 (106.0–242.0)	141 (104.0–189.5)	0.11
Glucose pre-transplant*	95.4 ± 32.4	95.7 ± 10.5	100.1 ± 49.5	92.6 ± 20.5	0.31
Glucose post-transplant	92.1 ± 19.9	94.6 ± 17.3	90.3 ± 20.77	92.1 ± 20.3	0.66
Transplant related					
Donor type, living†	107 (26.6)	9.0 (18.8) ^a^	27.0 (20.6) ^a^	71.0 (36.4) ^b^	<0.01
Number of hospitalizations**	1.28 ± 1.3	1.69 ± 1.7 ^a^	1.40 ± 1.4 ^a^	1.10 ± 1.1 ^b^	<0.01
DGF†	191 (51.3)	30 (63.8) ^a^	74 (56.5) ^ab^	87 (44.8) ^b^	0.02
PTDM†	92 (24.6)	13 (27.1) ^ab^	45 (34.4) ^a^	34 (17.4) ^b^	<0.01
Acute Rejection†	83 (22.2)	13 (27.1)	29 (22.1)	41 (21.0)	0.66
MDRD 3 months**	42 (28–55)	33.5 (20.2–48.0)^a^	37.0 (24.0–50.0)^a^	48.0 (35.5–60.0)^b^	<0.01
MDRD 6 months**	45 (35–58)	39.5 (27.2–56.7)^a^	42.0 (32.5–52.2)^a^	49.0 (30.8–60.0)^b^	<0.01
MDRD 12 months**	49 (37–60)	39.0 (23.0–60.0)^a^	46.0 (35.0–59.0)^b^	52.0 (40.0–60.0)^c^	<0.01
Prednisone†	372 (100)	48 (100)	126 (96.2)	189 (96.9)	0.18
Cumulative Prednisone dose (mg)**	21.93 (17.3–25.4)	21.3 (14.6–25.0)	21.6 (16.5–24.6)	22.1 (15.5–25.8)	0.74
Tacrolimus†	298 (80.1)	40.0 (83.3)	96 (73.3)	156.0 (80.0)	0.13
Cyclosporine†	69 (18.5)	4.0 (8.3)	28.0 (21.4)	31.0 (15.9)	0.16

SD = standard deviation, BMI = body mass index, HCV = hepatitis C virus, CMV = cytomegalovirus, HDL = high-density lipoprotein, LDL = low-density lipoprotein, DGF = delayed graft function, PTDM = Diabetes Mellitus post-transplant, MDRD = modification of diet in renal disease.

*ANOVA with Bonferroni post-hoc test

**ANOVAlog

†Chi-Squared.

P values were computed using χ2 or ANOVA, followed by post hoc multiple comparison tests (residual analysis or Bonferroni tests, respectively), as appropriate. Analyses are represented by superscript letters (a,b,c); different letters indicate difference between the groups at P < 0.05

## Results

**Fig 1:**
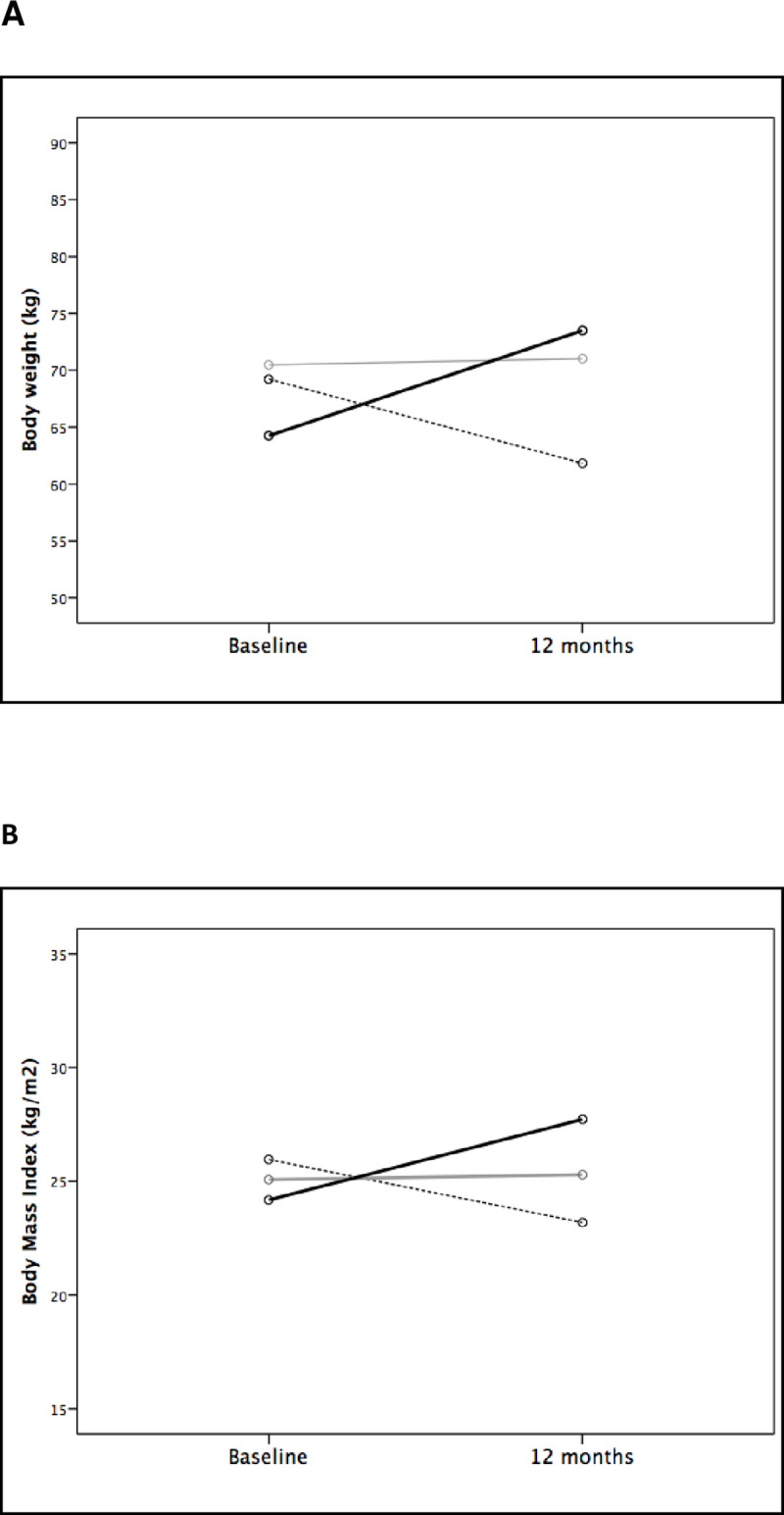
Body weight (A) and body mass index (B) variation from baseline to 12 months of follow-up according to body weight changes at 12 months (dashed line: weight loss >5%; grey line: stable weight (variation between -5% to +5%); and black line: weight increase >5%).

Out of the 756 patients submitted to kidney transplantation between January 2006 and July 2013, 445 were evaluated. 71 were excluded (<18 years old, n = 1; PTDM, n = 4; and incomplete weight data, n = 66), leaving 374 kidney transplant recipients remaining for inclusion (Table 1). There were 181 (48.4%) female patients, 334 (89.3%) with white ethnicity, and the mean age was 44.4 ± 12.8 years. In relation to the etiology of renal disease, 128 (34.2%) patients had undetermined etiology, 106 (28.3%) had hypertension, 61 (16.3%) had glomerulonephritis, 53 (14.2%) had polycystic kidney disease and 26 (7%) had other causes. The dialysis type was hemodialysis in 351 (93.9%) patients, with a median duration of 2.66 (1.33–5.16) years. The mean BMI pre-transplant was 24.7 ± 4.1 kg/m^2^; 35 (9.9%) patients were classified as obese; 119 (33.6%) as overweight; 187 (52.8%) as normal weight; and 13 (3.7%) as malnourished. After one year of follow-up, the mean BMI was 26.2 ± 5.0 kg/m^2^; 61 (17.3%) patients were classified as obese; 133 (37.8%) as overweight; 148 (42.0%) as normal weight; and 10 (2.8%) as malnourished. The Fig 1 shows the variation in body weight and body mass index from baseline to 12 months of follow-up post-transplant.

Weight gain was observed in 272 (72.7%) patients, and the average gain was 7.12 ± 5.9 kg. Among patients with weight gain, 195 (71.6%) had an increase of at least 5% in body weight, and 117 (43.0%) increased at least 10% from the baseline.

Patients were separated into different groups based on respective weight variation at one year of follow-up. Of the total sample, 195 (52.1%) had weight gain >5%, 131 (35%) presented a minor weight change (between 5% gain to 5% loss), and 48 (12.8%) patients lost >5% of their body weight. Table 1 shows demographic, anthropometric, clinical, and biochemical measures, according to these three groups. Patients who gained more than 5% of body weight were younger, more frequently female, had a lower body weight at baseline, and were less commonly CMV positive. In addition, the >5% gain weight group more frequently received a kidney from a living donor, had a lower need for hospitalization, lower frequency of DGF and PTDM, and higher eGFR at all times than the other groups.

A multivariable model was built, with >5% gain of body weight as the dependent variable and age at transplant, sex, pre-transplant weight, PTDM and rate of hospitalization as the independent variables (Table 2, Model 1). Female gender remained a risk factor for weight gain, while increased age at transplant, higher pre-transplant weight and number of hospitalizations were protection factors against weight gain. In this model, PTDM was not associated with weight variation post-transplantation. As the factors of receiving a kidney from a living donor, DGF and hospitalization rate are closely related, these three variables were not included in the same model. Separate models were constructed, exchanging hospitalization rate for the other two variables. The model including DGF showed similar results; however, DGF was not associated with weight variation and PTDM diagnosis was associated with protection against weight gain (Model 2). When living donor was included in the model (Model 3), female gender and pre-transplant body weight retain similar associations. The factor of living donor was associated with an increased risk for weight gain, and PTDM was a protection factor. In this model, transplant age lost the association with weight gain by more than 5%. The model fitting statistics for each of the model using the Hosmer and Lemeshow test is model 1 (including number of hospitalizations): 6.860 (chi-squared), 0.552 (significance); model 2 (including DGF): 6.749 (chi-squared), 0.564 (significance) and model 3 (including living donor): 12.580 (chi-squared), 0.127 (significance).

**Table 2:** Multivariable logistic regression analysis for weight gain >5% (dependent variable) in first year post-transplant.

	Multivariable regression	β (95% IC)	P value
Model 1	Gender (female)	1.681 (1.06–2.66)	0.03
	Pre-transplant weight	0.971 (0.95–0.99)	<0.01
	Age at transplant	0.982 (0.96–0.99)	0.04
	PTDM	0.617 (0.36–1.04)	0.72
	Number of hospitalizations	0.790 (0.66–0.93)	<0.01
Model 2	Gender (female)	1.712 (1.08–2.71)	0.02
	Pre-transplant weight	0.977 (0.95–0.99)	0.01
	Age at transplant	0.983 (0.96–1.00)	0.05
	PTDM	0.538 (0.31–0.90)	0.02
	DGF	0.664 (0.43–1.02)	0.06
Model 3	Gender (female)	1.733 (1.09–2.75)	0.02
	Pre-transplant weight	0.974 (0.95–0.99)	<0.01
	Age at transplant	0.989 (0.97–1.01)	0.26
	PTDM	0.540 (0.32–0.91)	0.02
	Living donor	2.122 (1.26–3.57)	<0.01

PTDM = Diabetes Mellitus post-transplant; DGF = Delayed Graft Function

## Discussion

In this retrospective cohort study conducted in a large tertiary care, university-affiliated hospital in southern Brazil, a high incidence (52.1%) of patients gained more than 5% of body weight in the first year after renal transplantation. There was a significant association between greater weight gain and youngest age, female gender, lower BMI pre-transplant, donor type (living), and lower number of post-transplant hospitalizations. In addition, DGF and PTDM were each observed at a lower frequency in this group, and eGFR was higher 3, 6 and 12 months post-transplant. Most importantly, both female gender and lower pre-transplant body weight were independently associated with 5% weight gain, after adjustments in different multivariate models. Receiving a kidney from a living donor and a lower need for hospitalization were also associated with higher weight gain, likely reflecting a healthier patient with a lower rate of post-transplant complications.

Weight gain is often observed in kidney transplant recipients, especially during the first 12 months post-transplant. In this study, after the first year of transplantation, 31.2% and 20.9% gained at least 10% and 5% of baseline body weight, respectively. These results are consistent with previous reports [15, 24]. Cashion et al., in a cohort study with 96 kidney transplant recipients, found a significant difference between mean weight (81.98 ± 1.81 kg vs. 84.30 ± 2.17 kg; P ≤0.01) one year post-transplant. According to the authors, this may be associated with younger age, income distribution and higher carbohydrate intake [15]. On the other hand, the Swiss Transplant Cohort Study (STCS, n = 792 kidney recipients) reported a very low body weight increase (0.7 ± 4.6 kg) one-year post-transplantation [25]. The differences between the Swiss cohort and this report may be related to cultural and behavioral factors, in addition to the fact that, in Brazil, the prevalence of obesity is higher than that observed in Switzerland [26]. A prospective observational study, aimed at comparing changes in body composition, lifestyle factors and metabolic responses in 31 adults who received a kidney from a living donor, showed a significant increase in body weight in these patients, at 3 and 12 months post-transplant (2.2 kg, P = 0.03 and 6.6 kg, P <0.0001, respectively). The authors noted that the weight gain was mainly visceral, and subcutaneous fat had a positive correlation with the occurrence of insulin resistance [27].

We identified two factors as independent predictors for weight gain of ≥5% from baseline to 12 months post-transplant in all multivariate models: female gender and lower pre-transplant weight. Other studies corroborated our findings. Female gender is a known risk factor for weight gain after renal transplantation [28, 29]. In a previous cohort study, weight gain one year post-transplant was significantly higher in women than in men [30]. Another report corroborating this finding, by Hap et al., with 62 kidney transplant recipients (38 men and 24 women), observed an increased incidence of overweight or obesity 24 months post-observation, and BMI increase was twice higher in women than observed in male recipients (1.90 ± 2.20 kg/m^2^ vs. 0.89 ± 1.85 kg/m^2^, P <0.001) [31].

Pre-transplant weight is an important factor to be considered when dealing with kidney transplant patients. Two recent studies reported that 29% and 38% of patients on the waitlist for renal transplantation were diagnosed as overweight or obese, respectively [32, 33]. In our study, the prevalence of overweight or obesity pre-transplantation was even higher (approximately 43.5%), reaching a prevalence of 55.1% at 12 months post-transplantation. However, the weight gain was lower in this subset of patients, as the group of patients that gained >5% of body weight had a lower pre-transplant BMI. Similar results were reported by Baum et al., who showed that an initial BMI above 25 kg/m^2^ predicted less weight gain 12 months post-transplant in a non-African American population [30]. The lower weight gain in overweight and obese transplant recipients could result from nutritional counseling that these patients may have received due to their increased BMI pre-transplant. Personalized nutritional counseling is routinely provided at our center of Nephrology of the Hospital de Clínicas de Porto Alegre to all patients during the immediate post-transplant period, and appointments with a registered dietitian are scheduled. However, not all patients attend the scheduled appointments, so it is unclear whether or not the lower weight gain observed in overweight subjects was due to nutritional interventions in our cohort. A possible nutritional intervention may also be an explanation for the opposite association between PTDM and post-transplant weight gain; however, this data was not collected in the present study. Furthermore, the prevention of weight gain is difficult in post-transplant subjects. A recently published randomized controlled trial, assessing whether an intensive nutrition intervention and exercise would reduce weight gain after kidney transplantation, reported results which suggested little correlation between nutritional intervention and post-transplant weight gain [34]. The intervention group (n = 19) received individualized nutrition and exercise counseling over twelve visits. The control group (n = 18) received standard nutritional care, based on guidelines, and four appointments with a dietitian. After 12 months, subjects’ mean body weight increased significantly by 4.6% (baseline: 78.0 ± 13.7 kg; 12 months: 81.6 ± 12.6 kg, P = 0.001), and was similar in intervention and control groups. In conclusion, the study did not show any advantage of intensive nutrition and exercise interventions for preventing weight gain, compared to standard nutritional management, in the first year after kidney transplantation. The authors did report that results suggested a relatively modest increase in body weight and fat in the standard nutritional care group [34]; however, the sample size was small and the study was not powered for definitive conclusions.

Age at transplant was a protection factor for weight gain in some of our study’s multivariate models. Renal recipients younger than 30 years showed greater weight increase after kidney transplantation than patients older than 50 years in previous studies [8, 35]. According to the National Health and Nutritional Examination Survey III (NHANES III), the impact of age and sex is consistent with patterns of obesity in the general population [36], as both factors are associated with higher prevalence of obesity.

The association of body weight gain and modifications in GFR is still controversial in the literature. An observational cohort study of 84 patients showed a negative correlation between changes in BMI and eGFR [37]. An increase in BMI during the first year after kidney transplantation caused a significant decline in renal function, particularly in the first 3 months post-transplant. On the other hand, a retrospective study of 165 renal transplant patients showed that patients who gained weight had increased eGFR (71.8 ± 20.3 vs. 77.4 ± 23.3 mL/min/1.73m^2^, p <0.01), and patients who lost weight had a decrease in renal function (66.4 ± 23.1 vs. 61.5 ± 24.5 mL/min/1.73m^2^, p <0.01) six months post-transplantation [38]. A third trial, including 1094 patients, lead to discrepant results, with the authors concluding that the association between changes in body weight and renal function varies according to the method used to estimate GFR, and future studies must be conducted to investigate this association [39]. In our cohort, DGF, which is classically implicated in lower GFR post-transplant, was not independently associated with post-transplant body weight changes.

To date, no other study has reported an association between the number of hospitalizations and weight variations after kidney transplantation. However, it is suggested that fewer hospitalizations are a consequence of lower rate of post-operative complications, and of better graft and patient survival [40]. Lower hospitalization rate may be a marker of better overall good heath, and so patients with less transplant-related complications would be hospitalized less frequently, and likely have a higher eGFR. In this sense, the association between number of hospitalizations and eGFR with higher post-transplant weight gain is likely not a cause-and-effect relationship. The same rationale may explain why recipients from living donors, which are less prone to complications and with a better overall state of health, gain more weight. Improvements in well-being may lead to greater appetite and, consequently, increased body weight.

This study has some limitations, mainly related to the retrospective design. We cannot be sure if the increase in body weight is due to fat accumulation or fluid overload, as variables such as body composition, dietary intake and physical activity were not evaluated, nor schooling and family income, which may be important factors associated with body weight post-transplant. Moreover, pre-transplant laboratorial data, collected from the dialysis centers, was incomplete. However, despite these limitations, this study succeeded in showing predictors of weight gain after renal transplant.

In conclusion, female gender and lower pre-transplant body weight were independently associated with weight gain of >5% in the first-year post-kidney transplant. Lower rate of hospitalization and donation from a living donor were also risk factors for this outcome. Patients with these characteristics should be closely monitored, in order to avoid excessive weight gain post-transplant. Large and prospective randomized clinical trials should be conducted to evaluate if nutritional preventive interventions are capable of minimizing body weight gain in higher risk populations.

## Supporting information

S1 Data (SAV)
